# Delay in arrival: lineage-specific influence of haemosporidians on autumn migration of European robins

**DOI:** 10.1007/s00436-022-07621-5

**Published:** 2022-08-24

**Authors:** Nóra Ágh, Tibor Csörgő, Eszter Szöllősi

**Affiliations:** 1 ELKH-PE Evolutionary Ecology Research Group, University of Pannonia, Veszprém, Hungary; 2 Department of Ecology, Molecular Ecology Research Group, University of Veterinary Medicine Budapest, Budapest, Hungary; 3 Behavioural Ecology Research Group, Center for Natural Sciences, University of Pannonia, Egyetem str. 10, 8200 Veszprém, Hungary; 4 Department of Anatomy Cell- and Developmental Biology, ELTE, Eötvös Loránd University, Budapest, Hungary; 5 Department of Systematic Zoology and Ecology, Behavioural Ecology Group, ELTE, Eötvös Loránd University, Budapest, Hungary

**Keywords:** European Robin, *Haemoproteus*, *Plasmodium*, Genetic lineages, Timing of migration, Body condition, Autumn

## Abstract

Haemosporidian blood parasites are widely used in evolutionary ecological research when exploring the effects of parasites on different life-history traits of their bird hosts. However, their roles in bird migration are less studied. If these parasites deteriorate the body condition of the birds strongly, they might negatively affect the whole migration phenology and the survival of the birds as well. In our study, we tested the relationships between infection for parasite genera (*Haemoproteus* or *Plasmodium*), the three most frequent parasite lineages and body condition (body mass, fat deposit), and the timing of autumn migration in the European Robin (*Erithacus rubecula*). We found that mean body mass and fat scores did not differ between parasitized and non-parasitized individuals, but infected juveniles arrived later than their non-infected counterparts. The difference in the arrival time of parasitized and non-parasitized birds was greater in the case of *Haemoproteus* infections. However, when we analysed the effects of the distinct parasite lineages separately, we found that prevalence of parasite lineages correlated with the body mass, fat storage, and timing of autumn migration of the birds in a different direction. Our results therefore emphasize the importance of testing the impacts of the different parasites individually, because possible lineage-specific effects on bird condition during migration might exist.

**Supplementary Information:**

The online version contains supplementary material available at 10.1007/s00436-022-07621-5.

## Introduction

Avian malaria and avian malaria-like parasites (*Plasmodium* and *Haemoproteus* spp.) are of special interest not only to veterinarians but also to ecologists. Because of the potential effects of these parasites on the whole life cycle of the birds, they have been in the focus of many previous studies (reviewed in Marzal 2012). However, it is difficult to explore the effects of avian malaria infections in wild bird populations. First, it is nearly impossible to detect individuals during the acute infection phase due to the inactivity of birds during this phase (Lapointe et al. 2012). So most knowledge on the effects of these parasites come from the chronic phase of infection or from experimental studies (e.g. Knowles et al. 2010; Palinauskas et al. 2018). Though detailed data about the mechanistic effects of these parasites are scarce (discussed later), some studies suggest that parasites negatively affect the physiological condition of the birds even in the chronic phase by destroying red blood cells. This results in reduced oxygen consumption rate and may manifest in slower flight distances of migratory birds (Valkiūnas 2005, but see Hahn et al. 2018). Furthermore, due to the increased oxidative stress, parasite infection may accelerate telomere shortening which ultimately affect the long-term survival of the host (Asghar et al. 2015).

It is important to note that there are more than 6000 lineages within the group of haemosporidians (Bensch et al. 2009) and their virulence potentially differs in different host species (Ilgūnas et al. 2019). Some lineages are host generalist infecting a broader range of host species, while some are host-specific (Waldenström et al. 2002; Dimitrov et al. 2010; Medeiros et al. 2014). Some studies found that generalist lineages reached lower intensities in their hosts than specialists did, because they are less adapted to certain host species but infected closely related host species more often than expected by chance (Hellgren et al. 2009). Generalist parasites might also be the most prevalent parasites in their compatible hosts (Hellgren et al. 2009; Drovetski et al. 2014) because these parasite species have strong immune evasion capabilities (Hellgren et al. 2007).

Different parasite lineages have different infectivity and pathogenicity on their avian hosts, leading also to various level of susceptibility of the hosts to these lineages (Dimitrov et al. 2015). Therefore, a number of studies have highlighted the importance of separately analysing the effects of different parasite lineages. Indeed, Ortego et al. (2008) found that male Lesser Kestrels (*Falco naumanni*) infected with a certain *Plasmodium* lineage (P-LK6) had less fledglings in their broods as the non-infected males, while in the Blue Tit (*Cyanistes caeruleus*), two closely related *Plasmodium* species (*P. relictum* and *P. circumflexum*) altered the mortality and recapture rate differently (Lachish et al. 2011).

Despite the relatively long known differences in the impacts and the transmission ability of these lineages, the exploration of how the effects of the lineages arise mechanistically has started recently. For instance, Hellgren et al. (2015) emphasized the importance of considering the origin of the lineages when studying the transmission and impacts of different avian malaria lineages. This is because they found that allelic variation in a specific parasite locus (MSP1, which is known to have a key role in the invasion of host red blood cells), might be linked to local differences in parasite virulence and host resistance.

Aželytė et al. (2022) found that during co-infection with two closely related lineages (P-GRW4 and P-SGS1; two lineages of the *Plasmodium relictum*), the lineage P-GRW4 started to disappear (and became undetectable with PCR methods) from the peripheral blood relatively fast when the intensity of P-SGS1 infection increased. Videvall et al. (2020) furthermore demonstrated that hosts infected with the highly virulent P-SGS1 showed a very high transcriptional response in genes which function primarily within the immune system or cell death regulation. However, the less virulent P-GRW4 caused much lower parasitaemia and just a minor transcriptome shift in the same direction as P-SGS1.

Similar differences were observed in the gene expression of the parasites, when Common Starlings (*Sturnus vulgaris*), i.e. a species with high tolerance for *Plasmodium* infection and a more susceptible species, the Common Crossbill (*Loxia curvirostra*) were experimentally infected with the same *Plasmodium* lineage. In the more sensitive host species, parasite expressed genes responsible for cell-invasion more intensely than in the parasite-tolerant host species. On the other hand, in the parasite-tolerant host species, parasite genes related to apoptosis or/and oxidative stress showed a higher expression level (Garcia-Longoria et al. 2020).

The impacts of the parasite genera and lineages may also differ during migration. In a long-distant migrant species, the Great Reed Warbler (*Acrocephalus arundinaceus*), the onset of autumn migration was delayed with increasing intensity of *Plasmodium* or mixed-genus infection, while *Haemoproteus* infection did not affect migration timing (Emmenegger et al. 2021). Shurulinkov et al. (2012) found that body condition of Yellow Wagtails (*Motacilla flava*) infected with *Haemoproteus motacillae* during spring and autumn migration was worse than non-infected individuals but no such correlation was found in the case of *Haemoproteus anthi* infection. Furthermore, parasites might alter the landscape movements of infected migrants during the refuelling period (e.g. Hegemann et al. 2018; Eikenaar et al. 2020) and as a result blood parasite infections may slow down the whole migration process.

In our earlier work (Ágh et al. 2019) where we had no possibility to sequence parasite lineages and did not identify parasite genera either, we found that infected juvenile Robins arrived later during autumn migration; however, prevalence had no effects on the actual body condition of the individuals. Here, we sequenced and incorporated parasite lineage data to the data used in Ágh et al. (2019). We studied whether a relationship exists between infection status of the birds and their condition and timing of migration.

## Material and methods

### Data sampling

We captured Robins at the Ócsa Bird Ringing Station (Central Hungary: 47°17′ N, 19°12′ E) using mist nests and following the Actio Hungarica capturing protocol (for details, see Csörgő et al. 2016). Robins sporadically breed and overwinter at this study site and are regular and common passage migrants during spring and autumn. The birds included in our study arrived from different areas of Europe; however, their breeding origin cannot be determined based on morphological characteristics (reviewed in Harnos et al. 2018). In our analyses, we included blood samples only from birds that were most likely under migration (samples were collected between 20th August and 5th November 2016; *N* = 403). Based on a long-term ringing data series in Hungary, we can state with high confidence that after 20th of August, the percentage of the transmigrate Robins is markedly increasing. Therefore, we can define this date as the start of the autumn migration period. We choose 5th November to be the end of the sampling period because after that date, we mainly detect overwintering individuals (Gyurácz and Csörgő, 2009 in the Hungarian Bird Migration Atlas).

Capturing and DNA sampling was conducted periodically so that 4 sampling days were followed by a 3-day break period. Blood samples were collected into 96% ethanol and kept at − 20 °C until analysis. We determined the age [*N*_juvenile_ = 345 (hatched in the year of capture), *N*_adult_ = 58 (older); Demongin 2016], measured wing feather length (the flattened maximum wing chord) and body mass of the individuals (see Ágh et al. 2019 for more details). We also estimated the subcutaneous fat deposition by fat scores (the fat scores normally range from 0 to 8, see Kaiser 1993); however, the birds in this study had fat scores only between 0 and 3. Arrival date was defined as the day of first capture of the birds at the study site (Harnos et al. 2015).

### Laboratory methods

The dataset used in this study was partially compiled by Ágh et al. (2019). In our current study, we added parasite lineage data to the previously used dataset where we had no possibility to sequence the lineages or to identify parasite genera and analysed the effects of the detected different lineages on their hosts. DNA was extracted with GeneAid Genomic DNA Mini Kit (Tissue) following the manufacturer’s protocol (Thermo Scientific™). Molecular sexing was performed to determine the sex of the birds and to check DNA quality (Suh et al. 2011). We excluded samples with unsuccessful amplification during molecular sexing (i.e. bad quality DNA samples, *N* = 7); therefore, we finally included 396 individuals (*N*_juvenile_ = 340 and *N*_adult_ = 56) in the analyses.

For the molecular detection of avian malaria and malaria-like parasites, we used a highly efficient nested polymerase chain reaction (PCR) method (Waldenström et al. 2004). To increase the sensitivity of the detection and to have enough DNA to the sequencing reaction, we used twice as much DNA as in our earlier study (Ágh et al. 2019). This resulted a higher overall prevalence (33.7%) in this study compared to Ágh et al. 2019 (14.9%). In all PCRs, both negative (ddH_2_O) and positive controls (samples that were previously confirmed to be infected) were included to control for possible contaminations and amplification failures during PCRs, respectively. However, neither negative controls nor positive controls ever showed contamination or amplification failures, respectively. To reduce the risk for false negatives, we screened negative samples twice for blood parasites.

To identify the different lineages, all samples with positive amplification were sequenced using the BigDye Terminator v3.1 cycle sequencing kit and sent to a capillary electrophoresis platform (Biological Research Centre, Hungary). Sequences were edited and aligned using the program BioEdit (Hall 1999) and identified to genus (*Haemoproteus* or *Plasmodium*) and lineage level by comparing sequence data with those of previously identified parasites reported in MalAvi database. Parasites with sequences differing by one nucleotide substitution were considered to represent evolutionary independent lineages (Bensch et al. 2004). We found 3 *Haemoproteus* and 12 *Plasmodium* lineages out of which 6 lineages were previously not reported (for GenBank accession numbers see Table 1). Out of these 15 lineages, there were only one frequent *Haemoproteus* (H-ROBIN1: *N*_adult_ = 13, *N*_juvenile_ = 39) and two *Plasmodium* lineages (P-LINN1: *N*_adult_ = 6, *N*_juvenile_ = 14; P-TURDUS1: *N*_adult_ = 6, *N*_juvenile_ = 28). Other lineages were present in the population in much less frequencies (prevalence ranged between 0.003 and 0.018). Therefore, the possible effects of the lineages on host physiology were analysed only for the frequent lineages (H-ROBIN1, P-LINN1, P-TURDUS1). Samples with multiple infections are difficult to identify to lineage level correctly due to the messy signal or the low signal intensity resulting in missing bases on the electropherogram. Therefore, mixed infections (only 4 cases) were omitted from the analyses. For prevalence data of the detected lineages, see Table 1.

**Table 1:** Prevalence of different avian malaria lineages. In the case of rare lineages, we add the age and sex of the infected individuals. “1Y” means juvenile and “1 + ” means adult. We found six new lineages (indicated in bold with the GenBank accession number) in low prevalence. The prevalence and the 95% confidence intervals were estimated with the Quantitative Parasitology 3.0 program

Genus	Lineage	*n* infected	Age/sex	Prevalence	95% *CI*
*Haemoproteus*	H-ROBIN1	52		0.131	0.1007–0.1688
	**H-Robin5 (MW554913)**	1	1Y male	0.003	0.0002–0.0145
	**H-Robin8 (MW554916)**	1	1Y female	0.003	0.0002–0.0145
*Plasmodium*	P-BT7	6	1Y male 1Y female	0.015	0.0067–0.0324
	P-GRW11	1	1Y female	0.003	0.0002–0.0145
	P-LINN1	20		0.051	0.0325–0.0766
	P-LK06	2	1Y female	0.005	0.0009–0.0184
	P-PBIP1	1	1Y female	0.003	0.0002–0.0145
	**P-Robin4 (MW554912)**	1	1Y female	0.003	0.0002–0.0145
	**P-Robin6 (MW554914)**	1	1 + male	0.003	0.0002–0.0145
	**P-Robin7 (MW554915)**	1	1Y female	0.003	0.0002–0.0145
	**P-Robin9 (MW554917)**	1	1Y female	0.003	0.0002–0.0145
	P-SGS1	7	1Y male 1Y female	0.018	0.0084–0.0361
	P-SW2	3	1 + female 1Y male 1Y female	0.008	0.0021–0.0274
	P-TURDUS1	34		0.086	0.0616–0.1183

### Statistical methods

We calculated the prevalence of *Haemoproteus* and *Plasmodium* parasites and the most common lineages in all age and sex categories with Sterne methods (Sterne 1954) with Quantitative Parasitology 3.0 (Reiczigel and Rózsa 2005) and compared them with Fisher’s test.

To test for differences in the average body mass of infected and non-infected individuals, we used a linear model with multiple comparison tests and compared the means of infected (*Plasmodium/Haemoproteus* or infected with a given lineage) and non-infected individuals in each sex and age group combination. Independent variables were the age, sex, and wing length.

In the case of fat categories 2 and 3, the sample size was too low (*N* < 5) after separating individuals by infection status. Therefore, to reach the best statistical fit and well-interpretable biological effects, we categorized the birds into two fat categories, those with no visible fat (score = 0, *N* = 200) and with fat (score = 1, *N* = 196). In the first genus-specific analysis, we assessed the relationship between fat scores (0/1) and infection category (non-infected vs. *Haemoproteus*-infected/non-infected vs. *Plasmodium-*infected individuals) using a generalized linear model (GLM) with binomial distribution and logit link function (Venables and Ripley 2002). In the second lineage-specific analysis, we repeated the comparison among the most common parasite lineages (non-infected vs. infected with H-ROBIN1 or P-TURDUS1 or P-LINN1). As sex and age classes did not differ statistically in fat scores, these two factors were not used as dependent variables in the model.

To study the possible differences in the average arrival time of the individuals, we used general linear models. Independent variables were sex and infection categories (non-infected vs. *Haemoproteus*-infected/non-infected vs. *Plasmodium*-infected) and their interaction. In a separate model, we analysed the effects of the three most common lineages (non-infected vs. infected with H-ROBIN1 or P-TURDUS1 or P-LINN1) on arrival time; the other independent variable was the sex. Because adults and juveniles arrived in different migration waves and to eliminate the effects of the unbalanced sample sizes, we built separate models for adults and juveniles when analysing the effects of parasites on timing of migration. All analyses were performed using R version 3.4.2. (R Development Core Team R 2017). For GLM we used “stats” package; for multiple comparisons, we used “multcomp” package, which contains *p*-value correction too (Hothorn et al. 2008). For the calculation of the effect size, we used the “effectsize” package.

## Results

The overall prevalence of *Haemoproteus* and *Plasmodium* species was 13.6% (95% *CI*: 10.58–17.39%; *N* = 54) and 19.9% (95% *CI*: 16.27–24.22%; *N* = 79), respectively. When comparing the two genera, the prevalence did not differ between age (*Haemoproteus*: *OR* = 1.92, *p* = 0.069; *Plasmodium*: *OR* = 1.31, *p* = 0.390) or sex categories (*Haemoproteus*: *OR* = 0.90, *p* = 0.773; *Plasmodium*: *OR* = 0.74, *p* = 0.220). For more details on sex and age group interactions, see the supplementary material (Table S1).

**Fig. 1:**
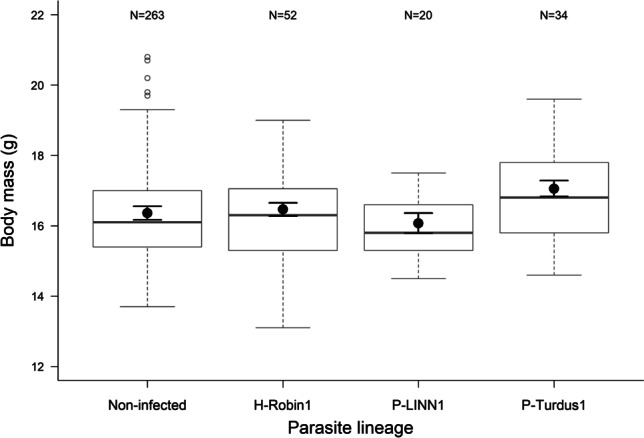
Body mass in relation to the most common parasite lineages in all age and sex groups merged. Box plots show the median, lower and upper quartiles and the whiskers representing data within the 1.5 × interquartile range and were calculated from the data. The black dots represent the mean body mass in each category with SE, estimated from the final model

There were no significant differences in the mean body mass between parasitized and non-parasitized individuals when compared non-infected individuals with *Plasmodium-*infected or *Haemoproteus*-infected individuals (*F*-value = 1.321, *p* = 0.270). However, P-TURDUS1-infected birds were heavier than non-infected individuals (estimate ± SE = 0.66 ± 0.224, *t* = 2.931, *p* = 0.004; Fig. 1; effect size: estimate [95% *CI*] = 0.52 [0.17, 0.86]). The interaction between age groups and different parasite lineages was non-significant; therefore, it was eliminated from the model (*F*-value = 0.807, *p* = 0.490).

**Fig. 2:**
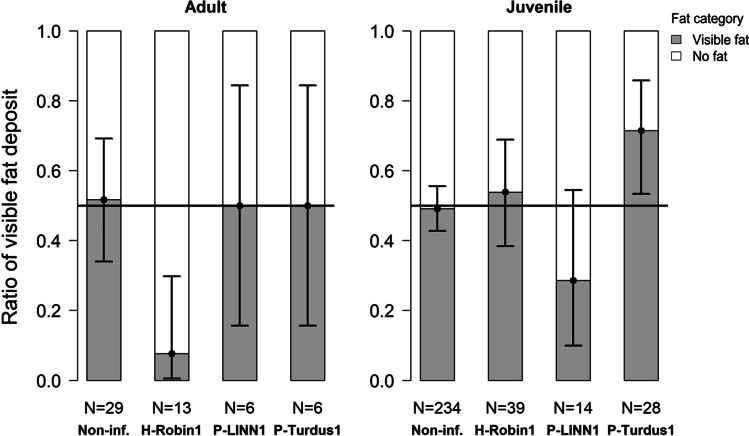
Ratio of having visible subcutaneous fat deposit in relation to different infection categories in adult (left) and juvenile (right) birds. Dots and whiskers show the estimated ratio of having visible fat deposit and the 95% confidence intervals, respectively, both calculated from the model

There was no significant difference in fat scores between *Haemoproteus*- or *Plasmodium*-infected and non-infected individuals (*OR* = 1.019, *p* = 0.797). However, we found that adult individuals infected with H-ROBIN1 had lower odds to have visible fat than non-infected adult individuals (*OR* = 0.084, *CI* = 0.005–0.423, *z* =  − 2.387, *p* = 0.017; Fig. 2; effect size: − 2.55 [− 5.52, − 0.75]). While, juveniles infected with P-TURDUS1 had higher odds to have visible fat than their non-infected counterparts (*OR* = 2.500, *CI* = 1.142–6.033, *z* = 2.190, *p* = 0.029; Fig. 2; effect size: 0.95 [0.13, 1.87]). In this respect, individuals infected with P-LINN1, juveniles infected with H-ROBIN1, and adults infected with P-TURDUS1 did not differ significantly from the non-infected individuals in the same age category.

*Haemoproteus*- and *Plasmodium*-infected individuals arrived later compared to non-infected ones, but only in the case of juveniles (estimates for adults see Table S3). The differences were greater in the case of *Haemoproteus* infections than for *Plasmodium*. There was no difference in arrival time when comparing individuals infected with *Haemoproteus* vs. *Plasmodium* (Table 2). If sexes were analysed separately, the greatest difference in arrival time was between non-infected and *Haemoproteus*-infected females (Table 2).

**Table 2:** Differences in mean arrival time between infected and non-infected juvenile individuals. The estimate values in days were calculated from the pairwise comparison for different infection statuses and sexes separately

Comparison	Estimate ± SE	*t*-value	*p*-value
Pooled data			
Non-infected-*Haemoproteus*	− 10.620 ± 3.020	− 3.517	0.001
Non-infected-*Plasmodium*	− 7.976 ± 2.501	− 3.189	0.004
* Haemoproteus*-*Plasmodium*	2.644 ± 3.557	0.743	0.733
Male			
Non-infected-*Haemoproteus*	− 4.890 ± 4.377	− 1.117	0.746
Non-infected-*Plasmodium*	− 8.060 ± 3.613	− 2.231	0.126
* Haemoproteus*-*Plasmodium*	− 3.170 ± 5.214	0.608	0.964
Female			
Non-infected-*Haemoproteus*	− 16.098 ± 4.160	− 3.870	< 0.001
Non-infected-*Plasmodium*	− 8.445 ± 3.459	− 2.442	0.076
* Haemoproteus*-*Plasmodium*	− 7.653 ± 4.839	− 1.528	0.435

**Fig. 3:**
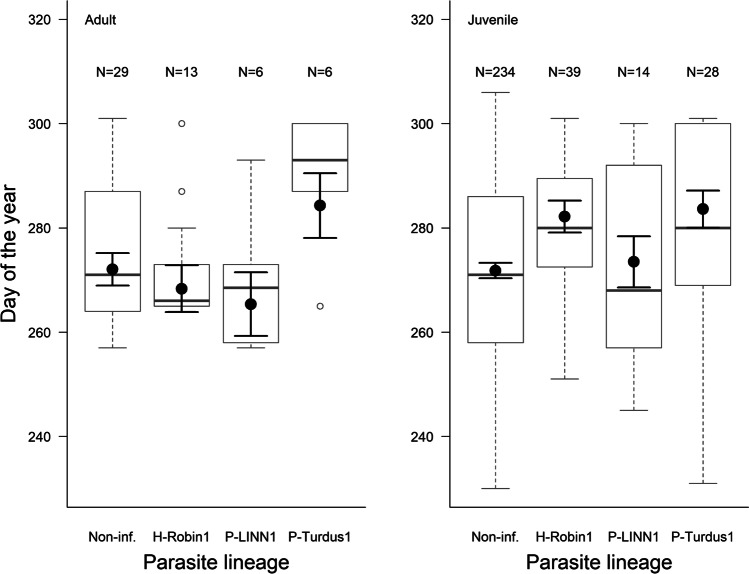
Arrival date in relation to infections with the most common parasite lineages separately for adults (left) and juveniles (right). Box plots show the median, lower, and upper quartiles and the whiskers representing data within the 1.5 × interquartile range. The black dots represent the mean arrival time in each category

Our lineage-specific analyses show that adults infected with P-TURDUS1 arrived marginally later than non-infected ones (estimate ± SE = 12.23 ± 6.197, *t* = 1.973, *p* = 0.054; Fig. 3; effect size: 0.85 [− 0.02, 1.71]); however, the sample size in the P-TURDUS1-infected group was very low (*N* = 6). On the other hand, juveniles infected with H-ROBIN1 (estimate ± SE = 10.37 ± 3.083, *t* = 3.363, *p* < 0.001; effect size: 0.57 [0.24, 0.90]) and P-TURDUS1 (estimate ± SE = 11.80 ± 3.567, *t* = 3.308, *p* < 0.001; effect size: 0.65 [0.26, 1.03]) arrived significantly later than non-infected ones (Fig. 3). The interaction between sex groups and parasite lineages was not significant; therefore, it was eliminated from the final model (*F* = 1.0291, *p* = 0.380).

## Discussion

Migratory birds cross different areas and habitats along their migratory routes and are exposed to a wide range of blood parasites (e.g. Waldenström et al. 2002). It was also hypothesized that they could potentially spread different parasite lineages to local resident species. However, later studies also showed that the number of migratory birds might not affect local parasite prevalence as prevalence was mostly influenced by macro-ecological patterns, like climatic differences among sites or vector communities (Shurulinkov and Ilieva 2009; Clark et al. 2016; Angeli et al. 2021).

Despite differences in the abundance and virulence of *Haemoproteus* and *Plasmodium* species (Scheuerlein and Ricklefs 2004; Astudillo et al. 2013), it is often difficult to detect any variation in the impacts of these parasites on the life history or morphological traits of the birds (e.g. Jenkins et al. 2015) because the effects of chronic infections are less pronounced (Valkiūnas 2005), and many times can be detected only on long term (Asghar et al. 2015). During experimental studies, the intensity of infection correlated with the rate of telomere loss (Asghar et al. 2015), affected the average haematocrit values, caused the blockage of blood vessels in the brain and resulted in higher mortality rate (Ilgūnas et al. 2019). However, to assess these questions, longitudinal data series or sometimes invasive sampling is needed.

The importance of analysing the effects of the different parasite lineages separately was supported by earlier studies. For instance in the House Martin (*Delichon urbicum*), the prevalence of the most common lineages showed opposite geographic patterns. The prevalence of *Haemoproteus* lineage (H-DELURB1) increased, while prevalence of *Plasmodium relictum* haplotypes decreased from North Africa to Europe. However, there were no lineage-specific differences in their impacts on body size, body condition, recapture rate, or survival of the individuals (van Rooyen et al. 2014). On the other hand, in other host and parasite species, differences were found among the lineages in the severity of the infection (Palinauskas et al. 2011), or in their effects on host fitness (Lachish et al. 2011; Shurulinkov et al. 2012).

In our study, the prevalence of two of the three most common lineages, P-TURDUS1 and H-ROBIN1 correlated with body condition, but in a different direction. Individuals infected with P-TURDUS1 was on average heavier than non-infected individuals, which was probably caused by the fact that P-TURDUS1-infected juveniles had a higher probability of having a fat deposit (having fat or not) than non-infected individuals. The interpretation of the results on P-TURDUS1 infection is complex. On one hand, it is possible that these individuals arrived at our study site in a better condition or we simply caught them at the end of their refuelling period. The fact that P-TURDUS1-infected individuals were captured later at our study site than non-infected individuals and individuals infected with H-ROBIN1 or P-LINN1 would suggest the latter.

On the other hand, adults infected with H-ROBIN1 had fat deposits less probably, but their mean body mass was similar to that of the non-infected individuals. It is possible that H-ROBIN1 infections caused a minimal delay in fat accumulation, but this was not manifested in differences in the body mass of the birds. However, it is important to note, that sample size for adult individuals was quite low, and the confidence intervals of the effect sizes were relatively wide (though the effect sizes were relatively high).

Hegemann et al. (2018) found previously in short-distance migrants (including Robins) that the landscape movements of haemosporidian-infected individuals increased, and these infected individuals stayed ca. three times longer at their stopover site to refill their fat reserves compared to their non-infected conspecifics. Another study on the long-distant migrant Great Reed Warbler found that migration timing was delayed and migration distances were shortened for individuals that were highly parasitized with *Plasmodium*, or with both *Plasmodium* and *Haemoproteus* parasites. To compensate their delay, infected Great Reed Warblers migrated significantly less far and had shorter resting durations (Emmenegger et al. 2021). However, Hahn et al. (2018) did not find any effects of low parasitemia on phenotypic attributes in this species during the preparation for migration. These results suggest that infection status may affect the movement capacity and the migration phenology of migratory birds, but infection status may only weakly associate with the actual body condition of the individuals as was discussed by the review of Risely et al. (2018). The short or medium-distant migrants, like Robins, probably follow other migration strategies, and perhaps, impacts and consequences of infections on migration phenology of these birds may also be different.

However, if the findings of Emmenegger et al. (2021) are also valid in our case, this would explain why H-ROBIN1-infected juveniles arrived later at our study site than non-infected individuals. This would also imply that *Haemoproteus* infections could slow down the migration process also in Robins. The small sample size of H-ROBIN1-infected adults (*N* = 13) would, however, explain why we did not detect similar relationship also in this age group.

The delay in arrival time was greater in females. Females occupy a subordinate position also during migration (Campos et al. 2011) and as a result often feed in less favourable habitats (Catry et al. 2004). Together with the negative effects of the infection, this may cause a higher delay in the arrival time of females. Unfortunately, we had too few recapture data about the studied individuals [*N*_non-infected_ = 43 (21 male, 22 female), *N*_infected_ = 23 (13 male, 10 female)], so we were not able to statistically analyse whether differences exist in the preparation time and efficiency between males and females and *Haemoproteus* infected and non-infected individuals.

It is also possible that later arriving H-ROBIN1-infected individuals simply came from more distant geographic areas and as a consequence of their longer journey, they arrive with more depleted fat reserves. This would also explain why these individuals arrived later. In this case, we would predict the prevalence of H-ROBIN1 infection to be higher in more northern populations; however, there is no information about the prevalence of the different lineages in other Robin populations to compare with.

Our and previous results therefore emphasize, that studying the effects of haemosporidian parasites on their hosts is particularly important during migration or during the dispersal period (Martínez-de la Puente et al. 2010; López et al. 2013; Emmenegger et al. 2020; but see Santiago-Alarcon et al. 2013; Emmenegger et al. 2018). If the number of infected birds increases for some reason and these birds arrive at the stop-over or wintering site in suboptimal time, this may even cause local population decline on the long term.

In addition to the three generalist and widely distributed lineages, we found some other, previously not detected parasites, which might be specialized to Robins. However, these parasites were present in such a low prevalence (*N*_12 rare lineages_ = 26; prevalence ranged between 0.003 and 0.018) that we were not able to analyse their effects on migration timing of the birds. We can only state that these rare parasites were detected mainly in the second half of the migration period (Fig. S1).

To summarize, individuals infected with haemosporidian parasites arrived later at the study site. The delay in arrival was more pronounced for *Haemoproteus* than for *Plasmodium* parasites. When focusing on individual lineages, we found that H-ROBIN1- and P-TURDUS1-infected juveniles arrived later than non-infected individuals. However, there were no differences between uninfected and *Haemoproteus/Plasmodium*-infected individuals in terms of fat storage or body mass. However, when we analysed the lineages individually, we found that TURDUS1-infected birds were heavier than non-infected individuals, adults infected with H-ROBIN1 had lower, while juveniles infected with P-TURDUS1 had higher probability to have visible fat than non-infected individuals.

Based on the magnitude of the effect sizes of the estimations and the biologically relevant differences found in timing of autumn migration of Robins, we stress the importance of studying the effects of haemosporidian parasites on migration phenology of this species. It is also important to note that we analysed only one migration period, and for some lineages, the sample sizes were so low that we can only carefully speculate the effects of the different lineages during autumn migration. To get a clearer picture on the effects of avian malaria during autumn migration and explore their effects on Robins on a broader scale, more data from different breeding populations and more detailed sampling of the migration populations (e.g. with radio-tracking, geolocators, see examples in Hegemann et al. 2018; Emmenegger et al. 2020) are needed.

## Supplementary Information

Below is the link to the electronic supplementary material.

ESM 1 (PNG 243 kb)

Supplementary file1 (TIF 1516 KB)

Supplementary file2 (DOCX 21 KB)
