# Supplementary figures and images for: Delay in arrival: lineage-specific influence of haemosporidians on autumn migration of European robins
Source: Parasitol Res. 2022 Aug 24;121(10):2831–40. doi: 10.1007/s00436-022-07621-5 (PMC9464164; doi:10.1007/s00436-022-07621-5)

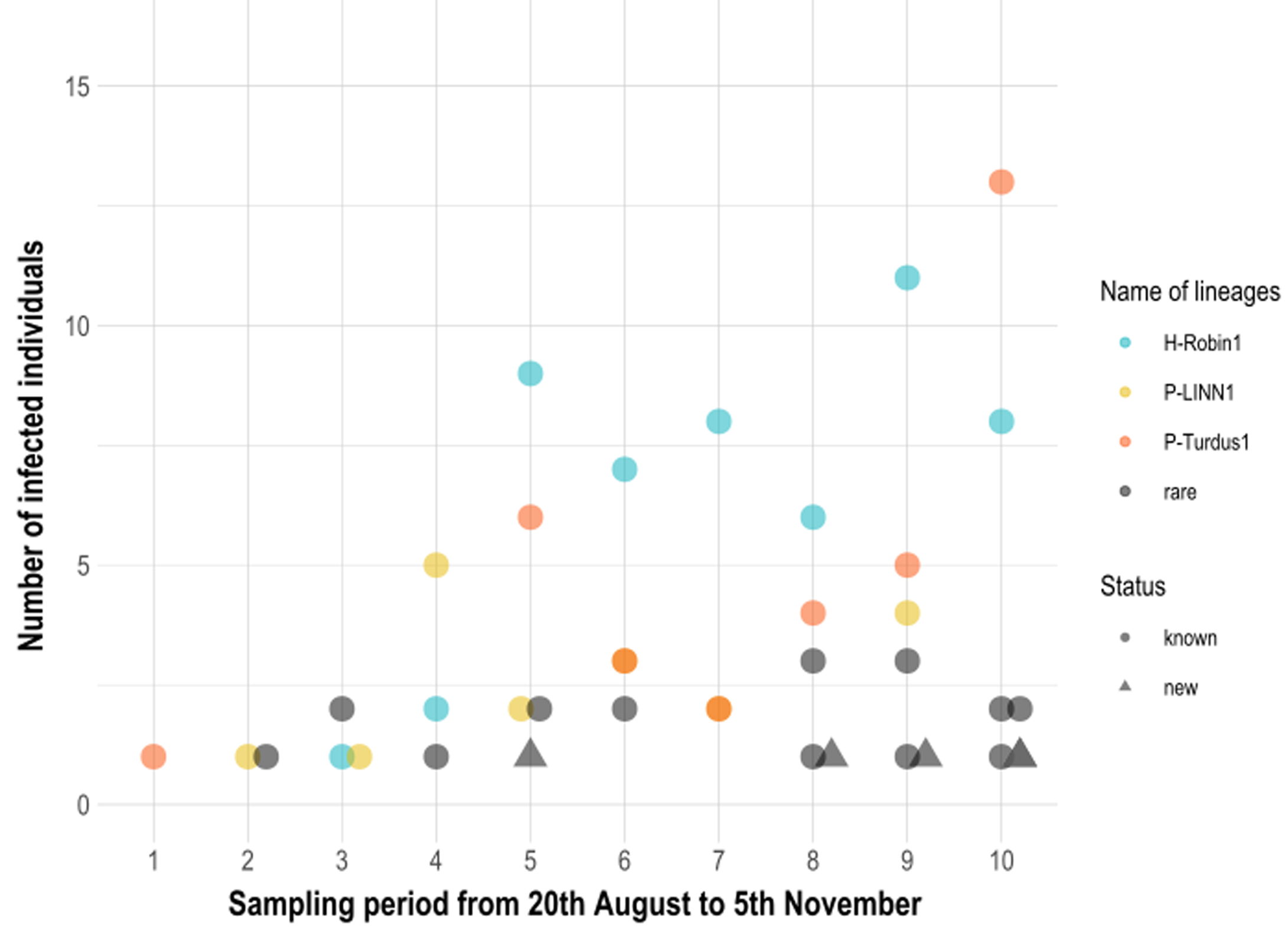

Supplement: Supplementary file 1 — (PNG 243 kb) [file 436_2022_7621_Fig4_ESM.png]

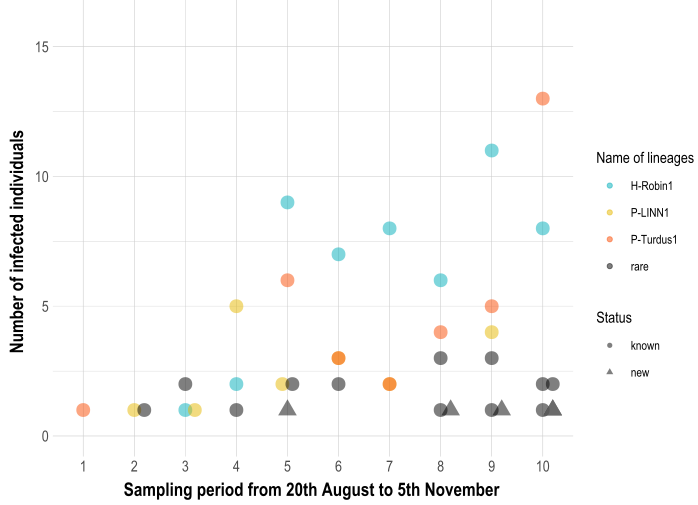

Supplement: Supplementary file 2 — Supplementary file1 (TIF 1516 KB) [file 436_2022_7621_MOESM1_ESM.tif]
