# Supplementary material for: Delay in arrival: lineage-specific influence of haemosporidians on autumn migration of European robins
Source: Parasitol Res. 2022 Aug 24;121(10):2831–40. doi: 10.1007/s00436-022-07621-5 (PMC9464164; doi:10.1007/s00436-022-07621-5)
Supplement: Supplementary file 3 — Supplementary file2 (DOCX 21 KB) [file 436_2022_7621_MOESM2_ESM.docx]

**Supplementary material**

Table S1: Prevalence of the two avian malaria genera in relation to age and sex group interactions.

Prevalence and its 95% confidence intervals are shown. χ^2^ and p-value belong to the Fisher’s test.

| Age:Sex | Genus | n (n infected) | prevalence | 95%CI | Test |
| --- | --- | --- | --- | --- | --- |
| adult:male | Haemoproteus | 26 (7) | 0.269 | 0.1286-0.4650 |  |
| adult:female | Haemoproteus | 30 (6) | 0.200 | 0.0909-0.3816 | OR= 0.75, p= 0.760 |
| juvenil:male | Haemoproteus | 175(19) | 0.109 | 0.0689-0.1649 |  |
| juvenil:female | Haemoproteus | 165(22) | 0.133 | 0.0868-0.1933 | OR= 1.23, p= 0.621 |
| adult:male | Plasmodium | 26 (4) | 0.154 | 0.0544-0.3436 |  |
| adult:female | Plasmodium | 30 (10) | 0.333 | 0.1773-0.5168 | OR= 2.14, p= 0.365 |
| juvenil:male | Plasmodium | 175 (30) | 0.171 | 0.1223-0.2338 |  |
| juvenil:female | Plasmodium | 165 (35) | 0.212 | 0.1537-0.2813 | OR= 1.24, p= 0.499 |

Table S2: Prevalence of the most common parasite lineages in relation to age and sex group interactions.

Prevalence and its 95% confidence intervals are shown. All values were calculated by the Quantitative Parasitology 3.0.

| Genus | Lineage | Age:sex | n (n infected) | prevalence | 95%CI |
| --- | --- | --- | --- | --- | --- |
| Haemoproteus | H-ROBIN1 | adult:male | 26 (7) | 0.269 | 0.1286-0.4650 |
|  |  | adult:female | 30 (6) | 0.200 | 0.0909-0.3816 |
|  |  | juvenil:male | 175 (18) | 0.171 | 0.1223-0.2328 |
|  |  | juvenil:female | 165 (21) | 0.127 | 0.0842-0.1872 |
| Plasmodium | P-LINN1 | adult:male | 26 (2) | 0.077 | 0.0139-0.2460 |
|  |  | adult:female | 30 (4) | 0.133 | 0.0469-0.2978 |
|  |  | juvenil:male | 175 (9) | 0.051 | 0.0260-0.0961 |
|  |  | juvenil:female | 165 (5) | 0.030 | 0.0121-0.0687 |
|  | P-TURDUS1 | adult:male | 26 (1) | 0.038 | 0.0020-0.1881 |
|  |  | adult:female | 30 (5) | 0.167 | 0.0681-0.3475 |
|  |  | juvenil:male | 175 (12) | 0.069 | 0.0390-0.1163 |
|  |  | juvenil:female | 165 (16) | 0.097 | 0.0598-0.1536 |

Table S3: Differences in mean arrival time between infected and non-infected adult robins. The estimate values in days were calculated from the pairwise comparison between infection status in the two sexes together and in a different model separately.

| Comparison | Estimate±SE | t-value | p-value |
| --- | --- | --- | --- |
| **Male and female together** |  |  |  |
| Non-infected - Hameoproteus | 3.865±4.790 | 0.807 | 0.699 |
| Non-infected - Plasmodium | -6.256±4.670 | -1.340 | 0.378 |
| Haemoproteus-Plasmodium | -10.121±5.527 | -1.831 | 0.168 |
| **Sexes separately** |  |  |  |
| **Male** |  |  |  |
| Non-infected - Hameoproteus | 5.038±6.581 | 0.766 | 0.921 |
| Non-infected - Plasmodium | -4.283±8.091 | -0.529 | 0.979 |
| Haemoproteus-Plasmodium | -9.321±9.012 | 1.034 | 0.798 |
| **Female** |  |  |  |
| Non-infected - Hameoproteus | 2.238±7.016 | 0.319 | 0.997 |
| Non-infected - Plasmodium | -5.229±5.953 | -0.878 | 0.877 |
| Haemoproteus-Plasmodium | -7.467±7.425 | 1.006 | 0.814 |

Figure S1: Appearance of the different *Haemoproteus* and *Plasmodium* lineages in relation to the sampling period. Each dot or triangle represents the number of individuals infected with a certain lineage. A “rare lineage” means a lineage was found in low prevalence in our sample (less than 3 individuals), A “new lineage” means a lineage previously not detected in Malavi (Bensch *et al.*, 2009)).
